# Self-limited corneal ectasia in a post-LASIK eye after cataract surgery: A case report

**DOI:** 10.1097/MD.0000000000035322

**Published:** 2023-10-27

**Authors:** Hao-Yun Chang, Wei-Ting Ho

**Affiliations:** a Division of General Medicine, Department of Medical Education, Far Eastern Memorial Hospital, New Taipei City, Taiwan; b Department of Ophthalmology, Far Eastern Memorial Hospital, New Taipei City, Taiwan; c School of Medicine, National Yang Ming Chiao Tung University, Hsinchu, Taiwan.

**Keywords:** case report, corneal ectasia, laser in situ keratomileusis, phacoemulsification cataract surgery

## Abstract

**Introduction:**

To present a case with a history of laser in situ keratomileusis (LASIK) developing central conic protrusion after phacoemulsification cataract surgery, which spontaneously resolved 5 months postoperatively.

**Patient concerns:**

A 55-year-old female who underwent myopic LASIK surgery 10 years ago presented to the clinic with bilateral cataracts and without ectasia. Following phacoemulsification cataract surgery and intraocular lens implantation in the right eye, the patient experienced a significant increase in spherical equivalent and corneal astigmatism.

**Diagnoses:**

Based on a central conic protrusion on topography examination, surgically-induced corneal ectasia was diagnosed.

**Interventions:**

Topical lubricants, corticosteroids, and serial follow-up with corneal topography.

**Outcomes:**

The corneal protrusion gradually resolved over a period of 5 months.

**Lessons:**

For post-LASIK patient who developed corneal protrusion following uneventful cataract surgery with a clear corneal incision, the clear corneal wound may have disrupted the biomechanical stability of the post-LASIK eye, compromising the peripheral stromal integrity. Additionally, postoperative inflammation could have contributed to corneal ectasia. Smaller clear corneal wounds or scleral tunnel entry during cataract surgery in post-LASIK eyes should be considered. Monitoring wound healing and using topical steroids can aid in achieving satisfactory outcomes and reducing the potential vision-threatening complications associated with corneal ectasia.

## 1. Introduction

Cataract surgery, especially phacoemulsification with foldable intraocular lens (IOL) implantation, is the most commonly performed ocular surgical procedure worldwide, owing to the nearly inevitable development of cataracts with age.^[1]^ Nevertheless, in eyes after laser in situ keratomileusis (LASIK), cataract surgery can be challenging due to changes in the corneal shape and thickness.^[2]^ While most studies focused on the inaccuracy of preoperative corneal power measurement as well as IOL power calculation, few studies mentioned about the impact of modern cataract surgery on the corneal biomechanical stability in post-LASIK eyes. Herein, we presented a case of a patient with a history of myopic LASIK who developed postoperative corneal ectasia following uneventful cataract surgery, which subsequently resolved spontaneously.

## 2. Case report

**Figure 1.**
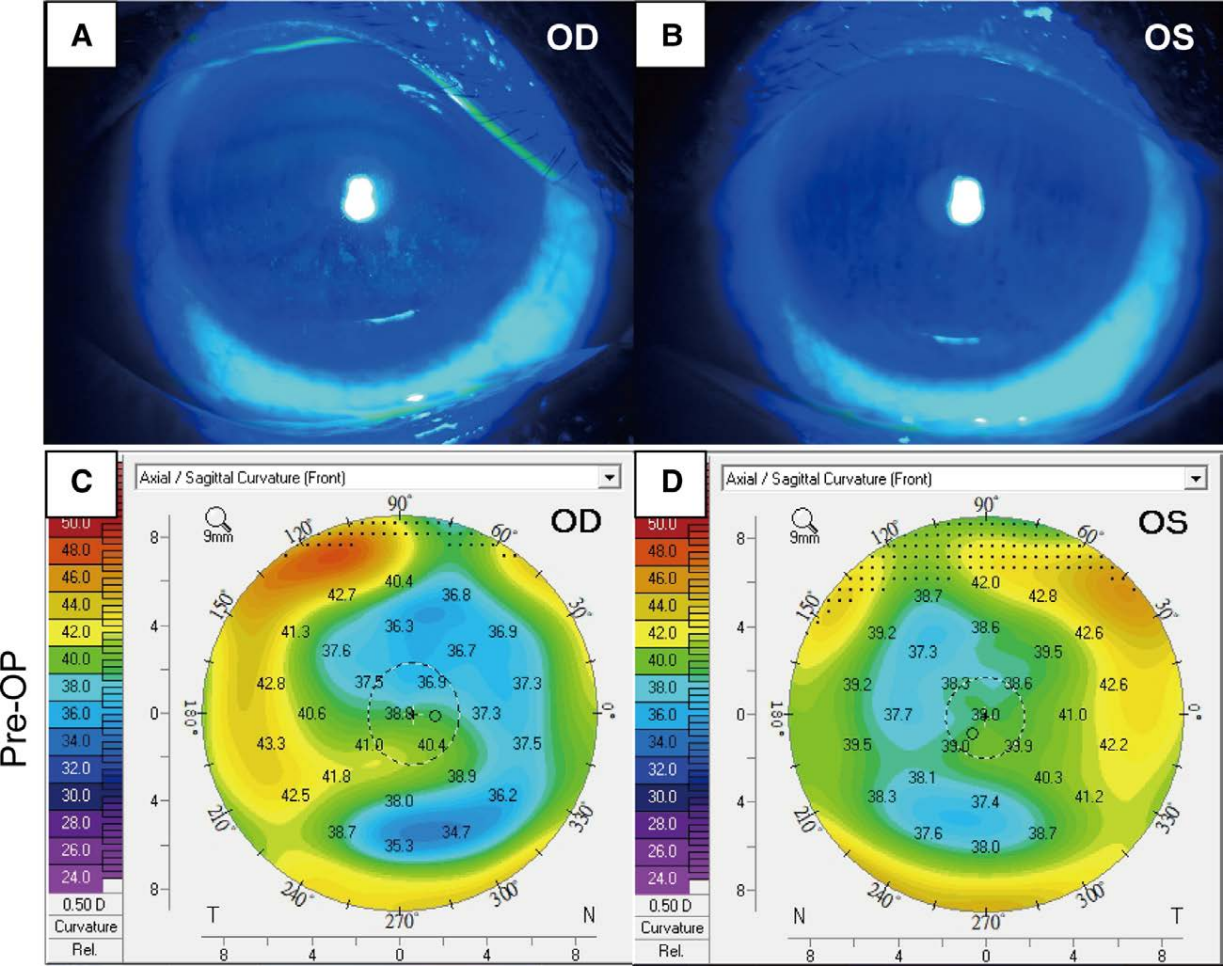
Preoperative corneal fluorescein staining and topography examinations demonstrated punctate staining due to dry eye disease (A and B), while no evidence of corneal ectasia in either eye was noticed (C and D).

A 55-year-old female with a history of myopic LASIK surgery in both eyes over 10 years ago and no other systemic illness presented to our clinic with a complaint of progressive bilateral blurred vision. The patient corrected visual acuity (CVA) was 20/50 in the right eye and 20/30 in the left eye, while the intraocular pressures in the right and left eyes were recorded as 13 mm Hg and 12 mm Hg, respectively. Slit lamp biomicroscopy showed bilateral cataracts, and fluorescein staining examination showed few punctate corneal erosions due to dry eye disease, while the LASIK flap margins were smooth (Fig. 1A and B). Fundus examination was unremarkable, and corneal topography revealed no evidence of ectasia (Fig. 1C and D).

**Figure 2.**
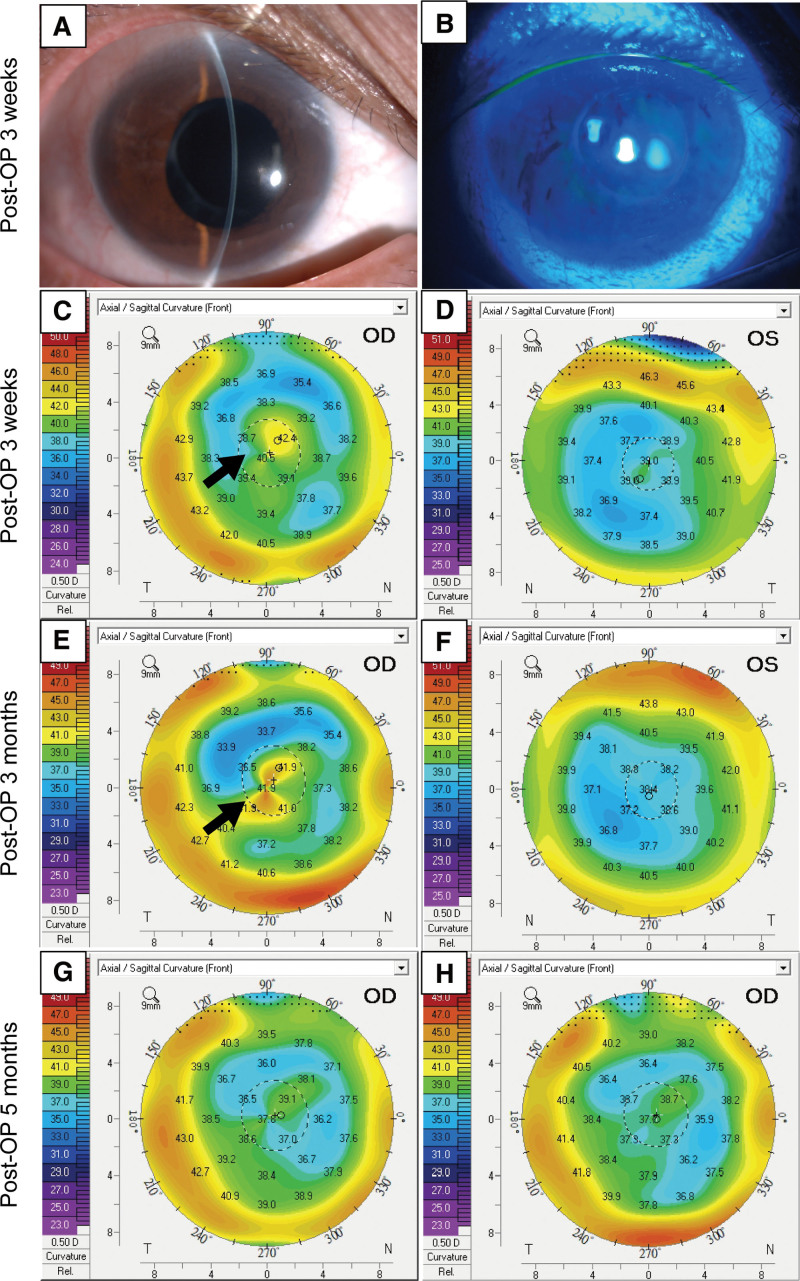
Self-limited corneal ectasia after cataract surgery. The external eye photographs of the right eye at the postoperative wk 3 showed no evidence of intraocular inflammation or inflammation at the laser in situ keratomileusis (LASIK) interface (A and B). Corneal topography at the same period demonstrated steepening of the central cornea in the right eye (C and D). The ectasia lasted for another 3 mo (E and F), but regressed under treatment with topical lubricants and corticosteroids 5 mo postoperatively (G and H). Arrows: conic protrusion in central cornea.

The patient subsequently underwent phacoemulsification cataract surgery and received a modified monofocal IOL (ICB00) implantation through a clear corneal incision of 2.75 mm in her right eye, which proceeded smoothly and without incident. At the first postoperative day, the spherical equivalent (SE) of her right eye was −0.625 D, with a CVA remained at 20/50. However, 3 weeks after the surgery, her SE increased to −4.75 D, and her corneal astigmatism increased from −1.0 D preoperatively to −2.5 D. The corneal topography of her right eye displayed central conic protrusion compared to her left eye (Fig. 2C and D). No signs of diffuse lamellar keratitis or interface fluid accumulation were found with slit lamp biomicroscopy (Fig. 2A and B). The patient was prescribed topical lubricants and corticosteroids. The 3^rd^ month postoperative corneal topography still showed central conic protrusion in the right eye (Fig. 2E and F), which resolved at the 5^th^ postoperative month (Fig. 2G and H). During her last visit (9 months postoperatively), the CVA recovered to 20/20, and her corneal astigmatism decreased to −0.5 D with a SE of −0.5 D.

## 3. Discussion

LASIK has gained popularity as a surgical technique for correcting myopia since its introduction in the 1990s. This procedure has a high patient satisfaction rate of up to 95% and is associated with a rapid recovery time and less postoperative discomfort.^[3,4]^ As a result, it has become the most frequently performed corneal refractive surgery worldwide. Despite the generally safe and effective nature of LASIK, corneal ectasia, a rare but devastating postoperative complication, has been reported to occur in 0.02% to 0.6% of patient.^[5]^ Studies have demonstrated that an abnormal preoperative topography indicative of keratoconus is the most significant predictor of post-LASIK ectasia.^[6]^ Other intrinsic risk factors worth considering include young age, low preoperative corneal thickness, low residual stromal bed thickness, and high myopia.^[6]^ Ectatic changes can manifest as early as 1 week postoperatively or even years later, although 50% of cases present within the first year and up to 80% present within 2 years.^[7]^

Our case underwent LASIK 10 years prior to her visit to our clinic, and the preoperative corneal topography showed no evidence of post-LASIK ectasia. However, the central cornea became protruded after a smooth phacoemulsification cataract surgery with IOL implantation through a clear corneal incision of 2.75 mm. One possibility is that the clear corneal wound during cataract surgery further disrupts the biomechanical stability of the post-LASIK eye. In the pre-LASIK eye, several forces play a role in the steady state of the cornea, including: The swelling pressure from hydrophilic stromal glycosaminoglycans that imbibe fluid; The centripetal force of the IOP; The tear evaporation, epithelial as well as endothelial barrier to counteract the swelling pressure of the stroma; and The cohesive forces between stromal lamellae.^[8]^ According to basic elastic shell models, ablation of the central corneal lamellae leads to protrusion and an increase in corneal curvature. However, the central ablation also results in peripheral stromal thickening, thus generating centripetal stress and central flattening.^[8]^ In our case, we made a clear corneal incision of 2.75 mm during cataract surgery, which may abrogate the resistance force against the central protrusion endowed by peripheral stroma. After the completion of stromal wound healing, the integrity of the peripheral stroma was reconstituted, thereby reforming the centripetal stress and central flattening. This clinical course exemplifies the crucial role of the peripheral stromal integrity in maintaining the flatten central corneal surface in the post-LASIK eye.

Although not a major contributing factor, the postoperative inflammation may play a role in the development of corneal ectasia in our patient. Ectasia has been associated with increased oxidative stress and several inflammatory markers, including MMP-9, IL-6, and TNF-α, and the imbalance of cytokines can disrupt corneal homeostasis and lead to keratoconus.^[9,10]^ Since an increase in IL-6 production has been observed in aqueous humors of patients who underwent cataract surgery,^[11]^ judicious application of topical corticosteroids may facilitate resolution of ectatic change in post-LASIK eyes undergoing further intraocular surgery.

## 4. Conclusion

As LASIK and cataract surgery are both widely performed ocular procedures, it is common that ophthalmologists may encounter patients who had LASIK in their younger age and underwent cataract surgery years later. To avoid corneal ectasia after cataract surgery, generating smaller clear corneal wound, such as 2.2 mm-incision, or entering the eye through scleral tunnel, may help to preserve the biomechanical integrity in post-LASIK eye. Additionally, carefully monitoring the wound healing process and application of postoperative topical steroids may help to achieve satisfactory surgical outcome and minimize the risk of this rare yet potentially vision-threatening complication.

## Author contributions

**Conceptualization:** Wei-Ting Ho.

**Data curation:** Hao-Yun Chang.

**Supervision:** Wei-Ting Ho.

**Writing – original draft:** Hao-Yun Chang.

**Writing – review & editing:** Wei-Ting Ho.
